# Studying the Emotional Response to Insects Food Products

**DOI:** 10.3390/foods10102404

**Published:** 2021-10-11

**Authors:** Michelangelo Serpico, Dominic Rovai, Kristine Wilke, Ruta Lesniauskas, Jeff Garza, Amy Lammert

**Affiliations:** 1 ESCP Business School–Turin Campus, Corso Unione Sovietica 218 bis, 10134 Torino, Italy; michelangelo.serpico@edu.escp.eu; 2 Department of Food, Bioprocessing and Nutrition Sciences, North Carolina State University, Raleigh, NC 27606, USA; drovai@ncsu.edu; 3 Garza Consulting, Grand Rapids, MI 49501, USA; kristine.wilke@the-gc.com (K.W.); jeff.garza@the-gc.com (J.G.); 4 Garza Consulting, Evanston, IL 60201, USA; ruta@the-gc.com; 5 Department of Food Science and Nutrition, California Polytechnic State University, San Luis Obispo, CA 93407, USA

**Keywords:** EsSense Profile^®^, edible insects, entomophagy, emotions, sustainability

## Abstract

Insects have been proposed as a sustainable food solution due to their environmental, nutritional, and socioeconomic value; however, in the western world, insects are viewed as disgusting. This research aimed to understand the acceptance of insect-based products in the US market by studying the emotional response to such. A survey of 826 consumers was conducted using (1) a modified version of the EsSense Profile^®^ questionnaire to capture the emotional response to pictures of different kinds of foods, (2) images to evaluate the influence of the presence or absence of non-visible insects in food products, (3) information about the environmental value of insects, and (4) socioeconomic demographics. Disgust was found as a barrier to product acceptance. Insect food products were positively correlated with the emotions of interest, understanding, daring, adventurous, and worried, and negatively correlated with the emotions satisfied, good, pleasant, happy, calm, warm, nostalgic, and secure. The influence of sustainability-related information on the emotional response to such products is complex and should be carefully considered.

## 1. Introduction

To date, food systems face the challenge of having to adapt to emerging social changes. Per capita demand for major commodities is expected to increase significantly by 2050, as the global population is predicted to grow to 9–10 billion people. In this regard, the FAO predicts a 60% increase in food demand and a 100% increase in meat demand [1]. Moreover, a recent Intergovernmental Panel on Climate Change (IPCC) report estimated the need for a 40 to 70% reduction of total greenhouse gas (GHG) emissions to maintain the average global temperature within a 2 °C increase above pre-industrial levels [2]. One of the most significant environmental challenges for food systems is reducing GHG emissions, as food systems contribute 20–30% of all man-made emissions. The most significant contributors are farming, agriculture, land-use change, and livestock, contributing around 14 to 18% of total emissions [3,4]. 

In this context, insects have been proposed by various authors as a potential sustainable food source, specifically as an alternative source to common animal protein sources [3,5,6,7]. Strong environmental benefits are associated with insect production in comparison to other types of livestock and include (1) specific emissions of greenhouse gases (GHGs) and ammonia during rearing are considerably lower; (2) their rearing requires fewer resources, including water and land; and (3) their efficiency of converting feed into protein is higher [8,9,10,11]. Despite their recognized value, barriers still exist to adopting insects as food, especially in the Western world [8,12]. Those barriers include production problems [3,8,13], legislative matters [5,8,14], and health-related issues [12,15]. However, one of the most important barriers to insect consumption is consumer acceptance. 

Some psychological factors have been found to be related to insects acceptance, such as food neophobia, disgust perception, and sensation seeking [16,17,18]. Disgust is recognized as one of the main barriers [18], as in the western world, insects are often seen as vectors of disease [19].

On the other hand, positive reactions to insects as food have been associated with beliefs that insects are nutritious and positive for the environment [20]. It may then be feasible to influence these beliefs through education [21,22]. For example, Mancini et al. [23] found that the provision of information regarding the nutritional, gastronomical, and environmental value of insects may lower their rejection and decrease disgust reactions.

However, insects still fail to be accepted by western consumers [24,25]. This suggests that a more complex perspective on food choice has to be taken into account, as suggested by some researchers who suggest a transdisciplinary approach involving both biological and social sciences to understand the various and complex interactions between shaping the acceptance of insect food products [26]. Most researches have strongly focused on psychological factors (such as disgust sensitivity and food neophobia) and the rational aspect of decision making (as shown by the attention given to education), while some important aspects regarding food acceptance have been overlooked, such as the role of emotions and sociocultural factors [25,27].

The importance of emotions has been recognized by researchers for a long time, and emotions are important in the decision making processes, affecting what we think (as for example, influencing our perception of risk), how we think about it (pushing for a more heuristic or systematic thinking), and implicitly shaping our decisions through behavioral responses [28]. More specifically, emotions and food consumption are two strictly related concepts, as emotions are both a consequence of food consumption and a powerful influence on our eating behavior and decision-making processes [29]. For this reason, studies evaluating emotions can provide information beyond traditional hedonic and preference measurements [30] and, therefore, can help to better understand consumer behavior and choice [31].

In the case of entomophagy, the relevance of emotions has been suggested by Onwezen et al. [32], who highlighted that affective factors could help better explain the acceptance of insects products, as consumers may rely more on affective processing given the novelty of the insects as food. Furthermore, Gmuer, Guth, Hartmann, and Siegrist [33], who studied consumers’ emotional responses to pictures of products containing insects, highlighted the importance of minimizing the negative emotional reaction (mainly related to disgust), and enhancing the positive experience and expectations of consumers. Le Goff and Delarue [34] evaluated facial expressions to study emotional responses to chips containing insects. They pointed out that consumers have strongly negative expectations of this kind of products, but the actual emotional response while consuming the product is significantly less negative, again suggesting the importance of building positive emotional expectations. Finally, Tan and House [27] emphasized that strong positive experiences toward entomophagy in Thailand, where it is traditionally consumed, suggest that familiarity and positive experiences seem to be an important factor in repeated consumption.

To increase consumers’ acceptance of insects foods, Looy and Wood [25] advised that a strategy should consider both the emotional and the rational dimension, integrating the understanding of consumer emotions with the provision of information and education about insects’ environmental values. This kind of strategy recognizes both the role of rationality (including the importance of values and goals) and emotions in shaping the formation of attitudes and in the translations of them into behavior [35]. However, some studies suggested that the emotional response to food products strongly depends on the product category considered [27]. Additionally, the practice of informing participants about the environmental value of insects may influence the emotional response [36].

Another proposed strategy to increase the acceptability of edible insects is the incorporation of powdered insects in familiar food products, as the incorporation of insects in familiar products may decrease neophobic reactions [37]. Additionally, research suggests that making insects non-visible may encourage consumption [27,33,38,39], possibly by minimizing the disgust related to the textural and optical attributes, which are the main elicitors of the aversive response [37,40]. Other researchers have also found that blindfolded participants tend to have difficulties in identifying insects by taste alone, further supporting this approach [41].

Incorporating insects in the non-visible form may help to remove culinary barriers related to the lack of knowledge regarding insect preparation [25,27,38]. Additionally, Tan and House [27] suggest the incorporation of processed insects in familiar food as a way to give importance to the fit of insects product in established eating practices, and therefore with the possibility of making insects product easier to be incorporated into existent diet patterns.

However, to the authors’ knowledge, no study has considered the practice of informing participants about the environmental value of insects in relation to the emotional response to different food categories incorporating non-visible insects. The objective of this research is therefore to understand the acceptance of different potential food products containing non-visible insects in the US market by studying emotions associated with such products, the influence of information given about the environmental value of insects, and relating these measurements to the willingness to try the products shown.

## 2. Materials and Methods

### 2.1. Recruitment and Screening

All online questionnaires were developed and completed using Red Jade software (Martinez, CA, USA). The questionnaires were distributed via social media (Facebook), email, and in-person referrals. No subject compensation was provided for participation. Prior to distribution, 51 people completed the questionnaire to evaluate the time required for questionnaire completion, and their responses were not considered for the analysis. Only data from subjects who accepted the informed consent, were over 18 years old, completed the entire questionnaire, and resided in the US were evaluated. Additionally, the time it took to participants to take the questionnaire was evaluated, and participants who completed the questionnaire too quickly were assumed not to have answered the questions seriously and were discarded, a practice that is common in the academic field [33]. A total of 1765 subjects completed the survey, but only 826 respondents met these criteria.

### 2.2. Experimental Design

Each participant first received a single questionnaire that was composed of three independent questionnaires. Questionnaire 1 (Age Screener) was a screener for age. Questionnaire 2 (Pictures Evaluation) was a questionnaire containing pictures of different product categories followed by a question regarding their emotions associated with the product categories, expected hedonic acceptance, and expected willingness to try these products. Questionnaire 3 (Demographics) was general sociodemographic questions.

#### 2.2.1. Age Screener

Participants provided information about their age (17 and under, 18–24, 25–34, 35–44, 45–54, 55–64, and 65 and over).

#### 2.2.2. Picture Evaluation

**Figure 1:**
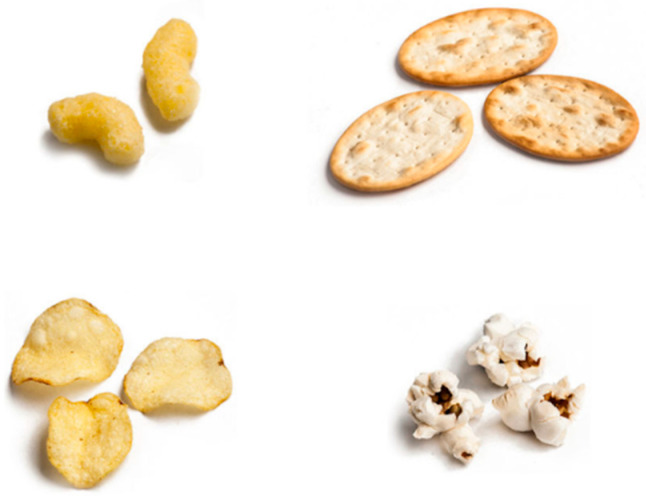
An example picture used to represent a Product Category. This image was used to represent the snack category. All pictures were taken by one of the authors.

Three different factors were evaluated to understand their effect on the emotional response of participants to the idea of food concepts that may contain insects: Product Category—different food product category images; Insect Presence—information about the presence or absence of non-visible insects in the products (as shown in Product Category images); and Sustainability Information—sustainability information regarding the environmental value of insects. The Product Category had five different levels, as five different pictures were shown representing the following product categories: (1) bread and pasta, (2) proteins supplemented products, (3) processed meat products, (4) sauces and dips, and (5) snacks. Figure 1 is an example of a Product Category image (all pictures were taken by one of the authors). The Insect Presence had two levels. The first option indicated the presence of non-visible insects in the product images shown, and the second option did not indicate the presence of insects shown in the images (please note that all images were the same, only the description changed). Sustainability Information also had two levels, as participants were given or not given sustainability information about the environmental value of insects. To decrease the amount of time spent on the questionnaire and decrease the cognitive fatigue of the participants, which is especially important when measuring emotional responses [42], while still ensuring the study of all main effects and the 2-way interactions, a D-optimal fractional factorial design with 17 treatments was created using Design Expert (Version 13, Stat-Ease, Inc., Minneapolis, MN, USA). Each participant, therefore, evaluated only 2 of the 17 treatments (Table 1). Treatment 6 and 17 (Table 1) are replicates to get an estimation of the true error in the design.

The Sustainability Information options were presented first, if required, by presenting three infographics that showed the environmental benefits of insects (greenhouse gas emissions, land usage, feed, and water requirements, respectively) compared to beef, pork, and poultry. These infographics were obtained from littleherds.org and used with the company’s permission. There was no information provided if the treatment did not require Sustainability Information.

The image for the selected Product Category in the treatment was presented next. Preceding the image was the following, only if the treatment was to include information about the presence of non-visible insects, sentence: “The following products contain INSECTS in a NON-VISIBLE FORM.” Beneath each picture was a brief description of its content; for example, the picture related to 7Sn having the following sentence: “This product category includes SNACK FOODS such as: CHIPS, CRACKERS, PUFFED SNACKS, and others.”

After each treatment image, the participant was asked to indicate their feelings with respect to products they typically consumed from the designated Product Category: “Considering this category of products, imagine eating a product that you frequently consume. Please select the words which describe how you FEEL RIGHT NOW. Select all that apply.” If Insect Presence Information were given, then the following phrase was added to the end of the first sentence: “[…] made with NON-VISIBLE INSECTS.”

The list of emotions provided was from the EsSense25 questionnaire [42], which was chosen for the following reasons: (1) it is suitable for application with a large number of participants, as it requires little time in gathering response; (2) it is easy to apply, as it does not require any specific instrumentation; (3) it is a non-specific questionnaire, therefore, it is suited for comparing products from different categories; (4) it has been already applied in online studies, and its application in this context has been proven suitable; (5) the number of emotional terms has been considered high enough to capture differences between different product categories, but not too high as to create cognitive fatigue of participants [43]; and (6) it has been proven comparably reliable to the EsSense Profile^®^, which has been successfully applied in a variety of situations and is extensively applied in the industry [44].

The term daring was added, taken from the EsSense Profile^®^ questionnaire [45], as it may be an important emotion when food products are made with insects. If the participants checked the term disgusted, another question was presented to them asking them to rate their feeling of disgust (Disgust Rating) on a 5-point scale (1 = Not Disgusted at All to 5 = Extremely Disgusted). Participants also indicated the expected overall liking of a product they frequently consumed considering the category shown in the image using a 9-point hedonic scale (1 = Dislike Extremely; 9 = Like Extremely) and their willingness to try using a 5-point scale (1 = Very Unwilling; 5 = Very Willing).

#### 2.2.3. Socioeconomic Demographics

Finally, participants answered sociodemographic-related questions, gathering information about their (1) gender, (2) residence, (3) ethnic background, and (4) educational level.

### 2.3. Data Analysis

#### 2.3.1. Screener and Socioeconomic Demographics

The age responses were recoded to three categories: 18–24 and 25–34 were recoded to Millennials (18–34), 35–44 and 45–54 were recoded to Generation X (35–54), and 55–64 and 65 and over were recoded to Baby Boomers (55 and over). The percentage of respondents (RStudio, Version 1.4.1717, RStudio, PBC, Boston, MA, USA) in each category was reported.

The gender (male; female; prefer not to answer), ethnic background (White or Caucasian; Hispanic or Latino; Black or African-American; Native American or American Indian; Asian or Pacific Islander; Other), education (Some High School; High School Graduate or Equivalent; Some College; Trade, Technical, or Vocational Training; Associate Degree; Bachelor’s Degree; Master’s Degree; Professional Degree; Doctorate Degree), and residence (San Luis Obispo County, CA; California; Other state in United States) were computed as a percentage of respondents (RStudio, Version 1.4.1717, RStudio, PBC, Boston, MA, USA) in each category for each socioeconomic demographic.

#### 2.3.2. Picture Evaluation

The emotional responses, collected as binary responses, were rescaled to percentages, to get a better reading of the results. Afterward, a model (RStudio, Version 1.4.1717, RStudio, PBC, Boston, MA, USA) was used to calculate the estimated percentages and means in respect to all responses except the Disgust Rating. ANOVA (RStudio, Version 1.4.1717, RStudio, PBC, Boston, MA, USA) was used considering three factors: (1) the treatment received, (2) the order in which the participants received the treatment, (3) the participant. For the Disgust Rating, the unadjusted mean was calculated, since the disgust question was given only to the participants who checked the term disgusted in the CATA evaluation. The data was analyzed using a one-way ANOVA (RStudio, Version 1.4.1717, RStudio, PBC, Boston, MA, USA), considering the treatment as a factor.

A multivariate principal component analysis (PCA) was completed (RStudio, Version 1.4.1717, RStudio, PBC, Boston, MA, USA) on the emotional responses, the Disgust Rating, and the expected liking and willingness to try responses to: 1) identify key similarities and differences among the treatments, and 2) illustrate the relationships between the emotional responses, the expected liking, the Disgust Rating, and the expected willingness to try. The estimated percentages of the emotional responses were used to calculate the principal components, excluding the emotion terms with a range of less than 10% across the treatments (free, tame, and aggressive), as these were considered not to have an impact on the emotional response. These three terms, the expected liking, the expected willingness to try, and the Disgust Rating, were included as supplemental variables. Expected liking and expected willingness to try were included as supplemental variables as the focus was given to the emotional responses. The supplemental variables and the 17 treatments presented in Table 1 were used to build the biplot, using the factor loadings for the selected emotional responses, the supplemental variables, and the factor scores for the 17 treatments.

The emotional responses, the Disgust Rating, the expected liking and the expected willingness to try responses, and the effects of the three experimental design factors (Product Category, Insect Presence Information, and Sustainability Information) were analyzed using ANOVA (RStudio, Version 1.4.1717, RStudio, PBC, Boston, MA, USA). The full model included the main effects and each two-way interaction of the experimental design factors. The ANOVA model (Design Expert, Version 13, Stat-Ease, Inc., Minneapolis, MN, USA) was fit using backward elimination, and model terms were removed based on significance at the 90% confidence level. The coefficient of determination (R^2^) was calculated with respect to each variable considered to measure the percentage of variance in the response explained by the design factors selected.

## 3. Results

### 3.1. Age Screener and Socioeconomic Demographics

The final study population (*n* = 826) was primarily female (87.89%), White or Caucasian (81.23%), had bachelor’s or higher degrees (72.59%), and resided in California (66.83%). The full demographic breakout can be found in Table 2.

### 3.2. Picture Evaluation

#### 3.2.1. Principal Component Analysis

**Figure 2:**
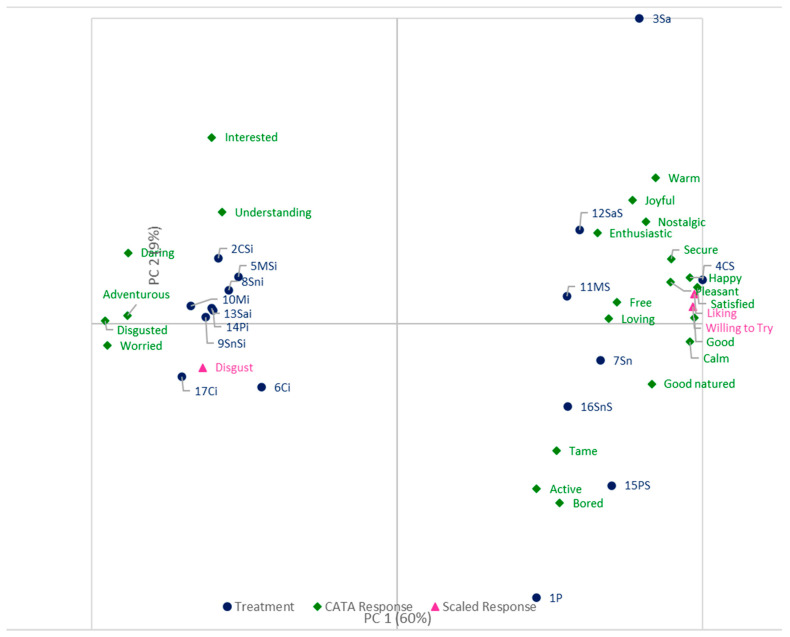
Biplot of the first two principal components, PC1 and PC2. The treatments are in blue, the CATA emotions from the EsSense Profile^®^ are in green, and the scaled responses are in pink.

The emotional responses, Disgust Rating, expected liking and expected willingness to try responses were used to extract the Principal Components, and the first two dimensions have been considered (PC1 and PC2). These two dimensions explain 69% of the variability among the 17 treatments; more specifically, PC1 explains 60% of the total variability, and PC2 explains 9%. PC1 and PC2 (data not shown) have been used to build the biplot (Figure 2). PC1 has the highest percentage of variability explained and is the most important dimension to understand the results. Treatments that are more associated with satisfied, the Expected Liking, good, the Willingness to Try, calm, happy, secure, pleasant, warm, good-natured, nostalgic, joyful, free, loving, enthusiastic, and tame tend to be positively correlated, and negatively correlated with disgusted, interested, worried, adventurous, the Disgust Rating, daring, understanding and aggressive.

The biplot (Figure 1) separates treatments for which the Insect Presence Information was given from treatments for which it was not given. When the Insect Presence Information was given, the treatments were more associated with interested, understanding, daring, adventurous, disgusted, and worried and higher ratings for Disgust. When Insects Presence Information was not given, the Treatments were more associated with warm, joyful, nostalgic, enthusiastic, secure, happy, pleasant, satisfied, good, calm, good-natured, tame, active, and bored, and higher ratings on the Liking and Willingness to Try scales. Furthermore, treatments 15PS, 16SnS, and 1P are more associated with tame, active, and bored and less associated with interested and understanding.

#### 3.2.2. Analysis of Variance

Results from the ANOVA for all responses are shown in Table 3. For each main factor and two-way interaction, *p*-values are presented (90% confidence level). Insect Presence Information, Product Category, and Sustainability Information are referred to as Insects, Product, and Sustainability in all figures and graphs. Each two-way interaction showed a significant effect for at least one response. The main effects and two-way interactions showing a significant effect (*p*-value < 0.10) were included in the regression model of each response. The regression models were used to build all the figures that will be presented below and to interpret the results.

**Figure 3:**
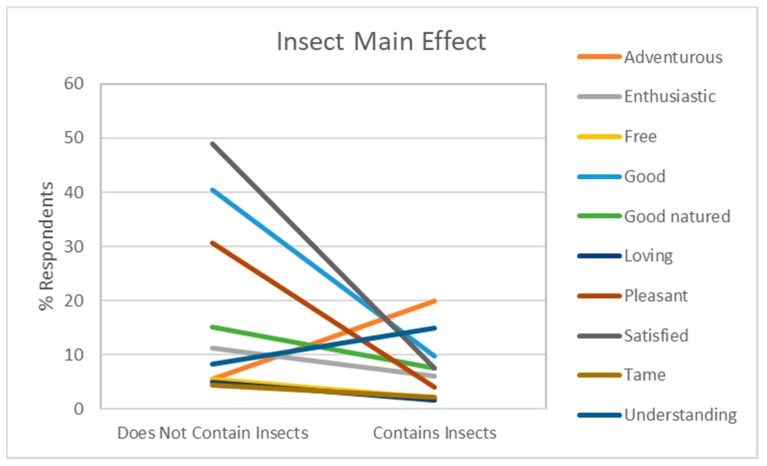
Effect of the Insect Presence Information on the emotional responses adventurous, enthusiastic, free, good, good-natured, loving, pleasant, satisfied, tame, and understanding. The y-axis indicates the predicted mean rating of Liking and Willingness to Try, and the x-axis is the presence or absence of Insect Information.

The Insect Presence Information did not have a significant effect on the emotions mild, wild, and aggressive, but did have a significant effect, independently from the Sustainability Information and the Product Category (i.e., no significant two-way interactions), for enthusiastic, free, good, good-natured, loving, pleasant, satisfied and tame (Figure 3). The Insects Presence Information decreased in the proportion of respondents indicating the emotions for satisfied, good, and pleasant. Conversely, the Insect Presence Information increased the proportion of respondents indicating adventurous and understanding. A lower average Liking and Willingness to Try was observed when the Insect Presence Information was given: Liking decreased on average by about 3 points (from 7.1 to 4.1) on a 9-point hedonic scale ((1 = Dislike Extremely; 9 = Like Extremely); Willingness to try decrease on average by about 1.5 points (from 4.2 to 2.7) on a 5-point scale (1 = Very Unwilling; 5 = Very Willing).

When evaluating the impact of Sustainability Information on products that did or did not contain insects, pleasant, adventurous, and understanding emotions were significant. For pleasant and adventurous, the impact of the Sustainability Information decreased for products that contained sustainability information: when Sustainability Information was given, the percentage of participants who checked the term “pleasant” decreased by around 7%, and the percentage who checked “adventurous” decreased by around 3.5%. The opposite was true for understanding, as the percentage of participants who checked this term increased by around 4.7%.

**Figure 4:**
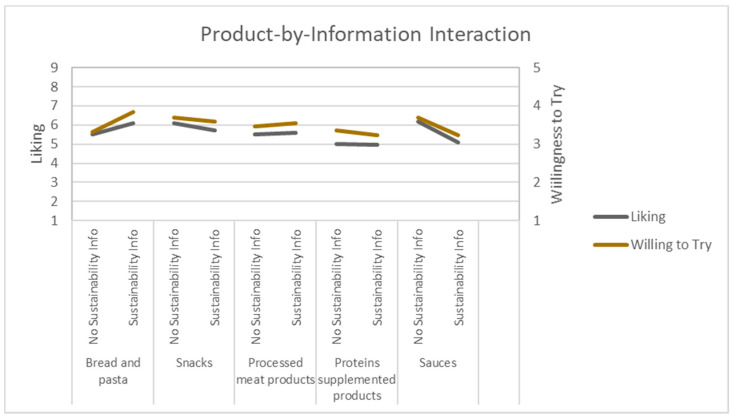
Effect of the interaction between the Sustainability Information and the Product Category on the expected liking (9-point hedonic scale, 1 = Dislike Extremely, 9 = Like Extremely) and expected willingness to try (5-point scale, 1 = Very Unwilling; 5 = Very Willing).

Liking and Willingness to Try responses had a significant interaction between the Product Category and the Sustainability Information (Figure 4). Results suggest a positive impact of the Sustainability Information on Bread and Pasta and a negative impact on Sauces.

**Figure 5:**
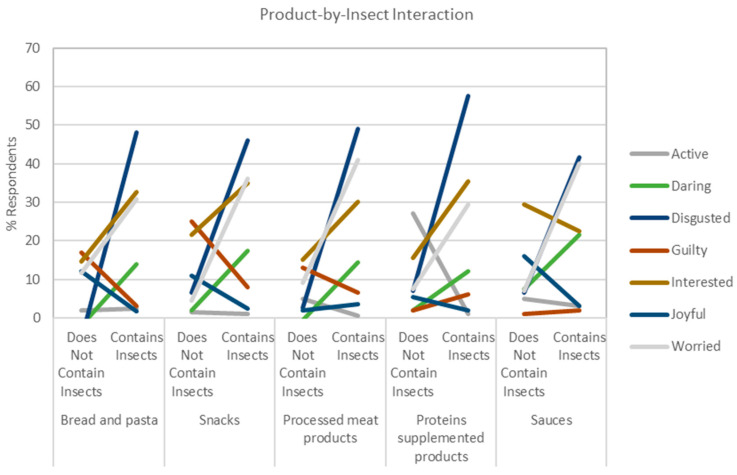
Effect of the interaction of the Insect Presence Information and the Product Category on Table 3. Effect of the interaction of the Insect Presence Information and the Product Category on the feelings bored, calm, happy, nostalgic, secure, and warm. The y-axis indicates the percentage of respondents checking the emotions, values that have been plotted using the regression model built from the ANOVA results, and the x-axis is the Insect Presence Information by each Product Category.

**Figure 6:**
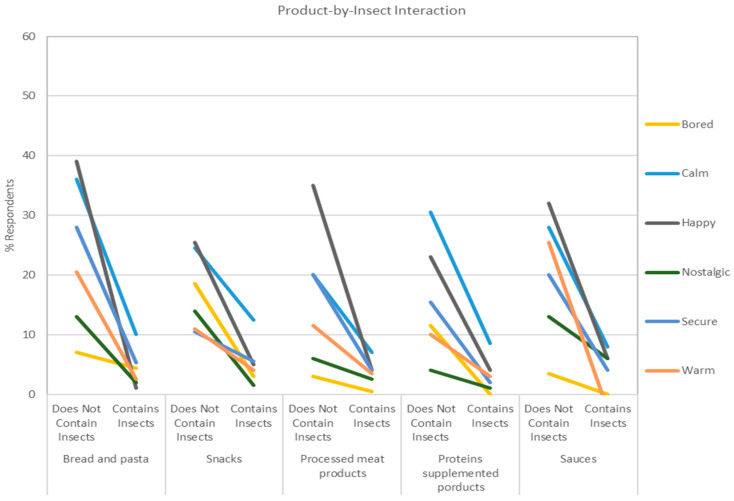
Effect of the interaction of the Insect Presence Information and the Product Category on the feelings bored, calm, happy, nostalgic, secure, and warm. The y-axis indicates the percentage of respondents checking the emotions, values which have been plotted using the regression model build from the ANOVA results, and the x-axis is the Insect Pres-ence Information by each Product Category.

**Figure 7:**
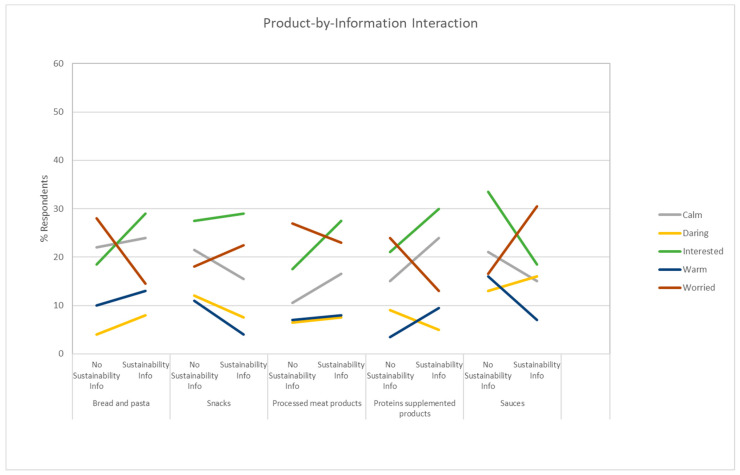
Effects of the interaction between the Sustainability information and the Product Category for the feelings calm, daring, interested, warm, and worried. The y-axis indicates the percentage of respondents checking the emotions, values that have been plotted using the regression model built from the ANOVA results, and the x-axis is the Insect Presence Information by each Product Category.

The Insects Presence Information had a significant interaction effect with the Product Category for the following responses with three different patterns of interactions. The pattern for daring, disgusted, interested, worried (Figure 5) indicated an increased percentage of respondents checking the emotions, with a particularly strong effect for disgusted and worried, but had the opposite effect for interested in the Sauce category. The pattern for guilty, joyful, and active (Figure 5) tended to be more strongly related to the Product Category. For example, for guilty, a strong decrease in the percentage of respondents checking this emotion is observed for Carbohydrate and Snacks, a less strong decrease for Processed Meat Products, and a slight increase for Protein and sauces. The pattern for bored, calm, happy, nostalgic, secure and warm (Figure 6) tended to decrease the percentage of respondents indicating the emotions, with a particularly strong effect for happy and calm. The effect for bored, nostalgic, and warm was stronger for Snacks and Protein, Carbohydrate and Snacks, and Carbohydrate and Sauces, respectively. Other interesting patterns have been also observed. In particular, the significant interaction effects between the Product Category and Sustainability Information for daring, interested, worried, calm, and warm (Figure 7).

The Insects’ Presence Information showed a significant interaction effect with the Sustainability Information in relation to both the disgusted and the Disgust Rating. For both responses, the Sustainability information showed an attenuation effect in respect to the Insects Presence Information, which tended to increase the percentage of participants checking the term disgusted and the mean for the Disgust Rating. When respondents were told that insects were present in the food products, the percentage of participants who checked the term “disgusted” increased by around 34% when Sustainability Information was given, and by 54% when no information was given, compared to the percentage of participants when insects were not present in the food products. Disgust Rating also increased by about 1.5 points on a 5-point scale (1 = Not Disgusted at All to 5 = Extremely Disgusted) when the Sustainability Information was not given, and of about 0.3 points when it was given.

#### 3.2.3. Treatments Comparison

Treatment 2CSi was further studied to understand the potential of marketing of insects in carbohydrate-rich products such as bread and pasta, as the Product Category Bread and Pasta was associated with an acceptable occurrence of the disgust feeling when the Insect Presence Information was given (Figure 5), and high Willingness to Try and Liking when the Sustainability Information was given (see Figure 4).

**Figure 8:**
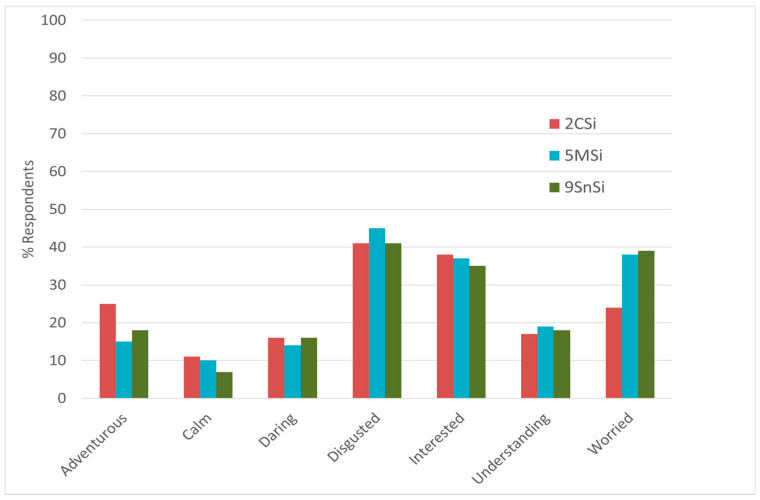
Estimated percentage of respondents considering the emotions for which either the Sustainability Information or the Product Category had a significant effect, and the treatments for which both the Sustainability Information and Insects Presence Information were given. Significance testing was not done across the treatments because the main focus of the study was to study the effects of the experimental design factors. Treatment abbreviations can be found in Table 1.

**Figure 9:**
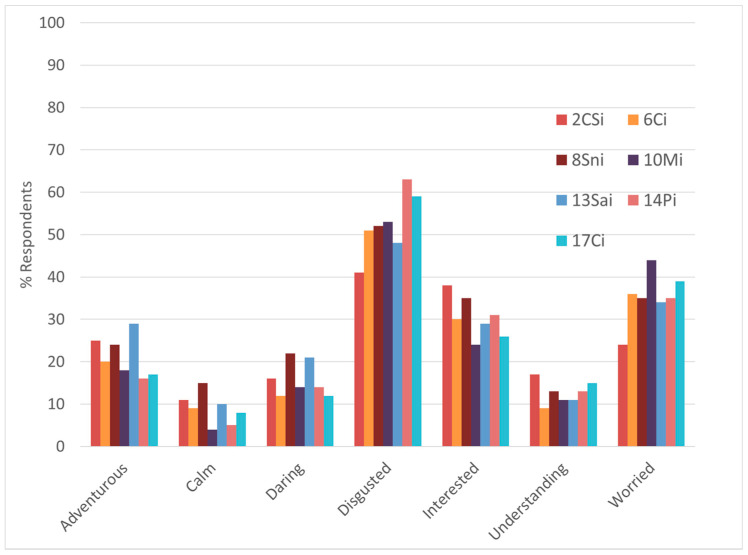
Estimated percentages of respondents considering the emotions for which either the Sustainability Information or the Product Category had a significant effect, and the treatments for which only the Insect Presence Information were given, and the treatment 2CSi. Treatment abbreviations can be found in Table 1.

First, treatment 2CSi was compared to the other treatments for which the Insect Presence Information was given, considering the estimated percentages of respondents for the emotions for which either the Product Category or the Sustainability Information had a significant effect (Figure 8 and Figure 9). For Figure 8 and Figure 9, only the emotions for which the incidence was more than 10% have been considered, as these were considered to have a decisive impact on the overall emotional response. From these figures, it may be observed that treatment 2CSi, in comparison with the other treatments for which the Insect Presence Information was given, showed good performances in respect to all emotions, including for disgusted, interested, and worried.

**Figure 10:**
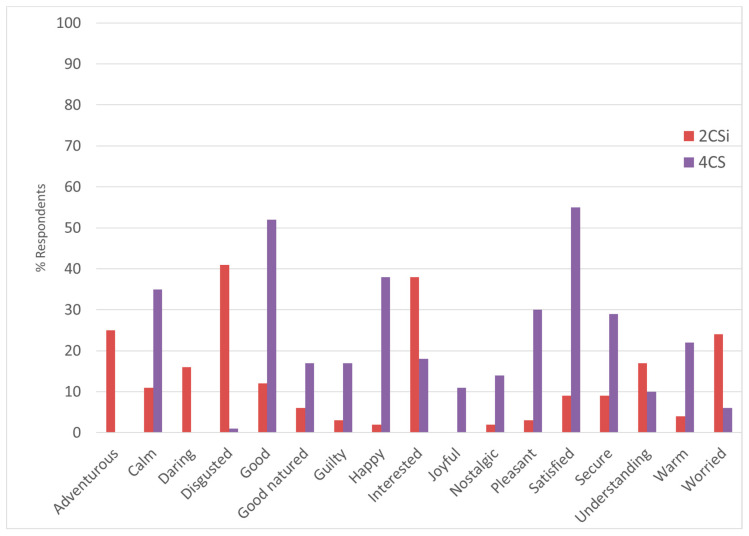
Estimated percentages of respondents considering all emotions and the Treatments 2CSi and 4CS. Treatment abbreviations can be found in Table 1.

Figure 10 presents the comparison between Treatment 2CSi and Treatment 4CS, i.e., the only treatment for which Bread and Pasta was used as Product Category and no Insect Presence Information was given; also here, only the emotions for which the incidence was more than 10% have been considered, as these were considered to have a decisive impact on the overall emotional response. From this figure, it can be observed that 2CSi performs better for adventurous, daring, guilty, interested, and understanding; on the other hand, 4CS performs better for calm, disgusted, good, good-natured, happy, joyful, nostalgic, pleasant, satisfied, secure, warm, and worried. Furthermore, 4CS had a higher Expected Liking and Willingness to Try. These results also suggest that if a product containing non-visible insects are marketed in a condition like 2CSi, acceptance of the product may be increased by improving the performance in respect to emotions such as calm, disgusted, good, good-natured, happy, joyful, nostalgic, pleasant, satisfied, secure, warm, or worried.

## 4. Discussion

The importance of the emotional reaction to insect products has been recognized by various authors [25,33]; this study suggests that all products containing insects in a non-visible form are associated with a set of specific feelings which go beyond disgust. Such insect-based products have been found to be positively correlated with emotions such as interested, understanding, daring, adventurous, and worried, and strongly negatively correlated with feelings such as satisfied, good, pleasant, happy, calm, warm, nostalgic, and secure. Moreover, this study confirms the role of disgust, showing that feelings of disgust are strongly associated with insect products which aligns with previous work done by Le Goff and Delarue [34] with facial expressions and Gmuer, Guth, Hartmann, and Siegrist [33] who studied the emotional responses of consumers to pictures of products containing insects.

The fact that the Insect Presence Information increased the occurrence of feelings such as adventurous and daring, and decreased the occurrence of feelings such as bored and tame is consistent with other works that found sensation seeking as a factor for insect acceptance [16,17,18,19], as well as research that proposed that a viable target for insect products could be consumers seeking novel and unique food experiences and adventurous eating [24,46]. However, insect products were strongly negatively associated with expected liking and expected willingness to try in this study, which was more associated with the positive emotions (satisfied, good, happy) negatively correlated with insect products. These results also suggest the need for creating more positive emotional expectations and a reduction in the negative emotional responses, as suggested by previous research [27,33].

This research also indicates that presenting insects in the non-visible form is not enough to increase acceptability, even though some authors have proposed such a solution as a way to remove disgust-related reactions and increase acceptance [6,37]. This research indicates that negative emotions like disgust and worried are associated with products containing non-visible insects as well, and that the presence of insects in a non-visible form still significantly decreases the expected liking and willingness to try such food products.

Thus, additional solutions need to be found for marketing insect foods. Various authors proposed the use of education to provide information about the sustainability of insects to increase insect-based food acceptance [21,22]. Results from this study highlight some important issues about the use of such information regarding food products containing non-visible insects. Education on the sustainability value of insects had a slight positive effect on disgust responses towards insect products, which is not consistent with previous studies on willingness to consume burgers containing non-visible insects, which suggested no effect of sustainability information on disgust reactions [36]. This may be due to differences in consumer values when it comes to answering a survey, compared to consuming an actual product where sensory characteristics may influence consumer attitudes. In addition, the study demographics between the two studies differed significantly. However, the low mean scores for expected liking and willingness to try products for which both the Sustainability Information and the Insect Presence Information was given suggest that more than just educational information alone should be considered. Emotions related to insect-based foods are complex and should be considered. For example, the emotions adventurous and pleasant decreased when Sustainability Information was presented, while the opposite is true for the emotion understanding. Moreover, significant interactions have been found between the use of sustainability-related information and the product form regarding various emotions, including daring, calm, interested, warm and worried, with effects strongly varying according to the specific Product Category.

The use of information had a positive impact on the expected liking and willingness to try of products such as bread, pasta, and meat processed foods, and a negative impact on products such as sauces (Figure 4). The use of sustainable information should be carefully considered as it could be negative in certain contexts. For example, if adventurous eaters were to be targeted, the emotions such as daring and adventurous need to be increased, the use of sustainability-related information, if combined with the incorporation of non-visible insects in snacks and protein-supplemented products such as protein bars, may be counterproductive.

This research suggests that the choice of the product form has a strong impact on the emotional reactions of consumers to products containing non-visible insects. Interesting interactions were observed in relation to active, guilty, joyful, disgusted, interested, and secure. For sauces, the incorporation of non-visible insects provoked a lower occurrence of disgust feelings with respect to other categories; however, there was also observed a decrease in interested and an increase of guilty, contrarily to other categories, and a lower decrease in bored. Carbohydrate-rich products such as bread and pasta were associated with a lower increase of disgust feelings as well; additionally, it was observed a slight increase of active and a strong decrease of guilty when associated with non-visible insects. Protein-supplemented products such as protein bars showed a lower increase of disgust feelings; however, the impact was overall negative, including a strong decrease in the occurrence of active, secure, and joyful, and an increase in the occurrence of guilty. For meat processed foods, interesting interactions were observed; this was the only category for which the presence of non-visible insects was associated with an increase of the occurrence of joyful; moreover, it was also observed a strong decrease for the emotion guilty, contrarily to protein supplemented products and sauces. For snacks, a very strong decrease in guilty was observed, even though this Product Category was also associated with a strong increase in disgusted, and a strong decrease in joyful and secure.

Considering treatment 2CSi, such a solution, compared to all other treatments for which the Insect Presence Information was given, showed better overall performances in respect to the most occurring feelings, including adventurous, calm, daring, disgusted, interested, understanding, and worried. Furthermore, comparing treatment 2CSi to treatment for which Insect Presence Information was not given (treatment 4CS), treatment 2CSi performed better for some emotions, including adventurous, daring, guilty, interested, and understanding; however, in respect to treatment 2CSi, the comparison suggests that the expected liking and the performance in terms of various emotions (calm, disgusted, good, good-natured, happy, joyful, nostalgic, pleasant, satisfied, secure, warm, and worried) need to be improved to successfully market products in such conditions.

Overall, such observations reinforce the need to carefully take into consideration the product form, highlighting the potential of incorporating non-visible insects in carbohydrate-rich products such as bread and pasta.

### Limitations and Future Research

The results from this study are limited to the population taken characterized by a high proportion of white females, highly educated, and mainly living in California; more specifically, females represented 87.89% of the sample, white people represented 81.23% of the total participants, and 66.84% of the participants resided in California. Therefore, further development is needed to understand the application of these results for a broader population. These results should be compared with physical product testing, as this study is limited to the use of pictures in an online environment. Moreover, the influence of different types of insects should be considered, as participants were given a general indication about the presence of non-visible insects without any detail on the species. Additionally, other methods of measuring emotions should be taken into consideration as this research only used a self-report questionnaire. Finally, the study of the emotional responses should be compared to a segmentation analysis to explore the potential different emotional responses by individual segments and to confirm the existence of a segment of consumers attracted by the novelty of insects.

## 5. Conclusions

The emotional response to foods containing non-visible insects goes beyond disgust, as they are positively correlated with feelings such as interested, understanding, daring, adventurous, and worried, and negatively correlated with feelings such as satisfied, good, pleasant, happy, calm, warm, nostalgic, and secure. Effective solutions to improve acceptance of these products and increase positive emotional expectation is needed, as in the current context, an overall low expected liking and willingness to try characterizing these products. The use of sustainability-related information for marketing insects is complex and may not be beneficial or positive. Careful consideration should be taken with the choice of the product form, as the impact is complex and varies in combination with the use of sustainability information and the final product form. From this point of view, these results suggest that a good starting point could be the use of sustainability information in combination with the incorporation of non-visible insects in products such as bread and pasta (treatment 5CSi).

## Figures and Tables

**Table 1:** The 17 treatments evaluated in this experiment. The Product Category was indicated by the first one or two letters (C for bread and pasta, M for processed meat products, P for protein supplemented products, Sa for sauces, and Sn for snacks). Sustainability Information was indicated by the letter S only if the information was provided in the treatment. The letter i indicated the Insect Presence Information only if the information was to be given. For the treatment designated 1P, the subjects saw a picture of protein supplemented products (P), with no Sustainability Information or Insect Presence Information, but for the treatment designated 9SnSi, the subjects saw a picture of snacks with both Sustainability Information and Insect Presence Information.

	Factor 1	Factor 2	Factor 3
Treatment Code	Product Category	Insect Presence Information	Sustainability Information
1P	Proteins supplemented products	No	No
2CSi	Bread and pasta	Yes	Yes
3Sa	Sauces	No	No
4CS	Bread and pasta	No	Yes
5MSi	Processed meat products	Yes	Yes
6Ci	Bread and pasta	Yes	No
7Sn	Snacks	No	No
8Sni	Snacks	Yes	No
9SnSi	Snacks	Yes	Yes
10Mi	Processed meat products	Yes	No
11MS	Processed meat products	No	Yes
12SaS	Sauces	No	Yes
13Sai	Sauces	Yes	No
14Pi	Proteins supplemented products	Yes	No
15PS	Proteins supplemented products	No	Yes
16SnS	Snacks	No	Yes
17Ci	Bread and pasta	No	Yes

**Table 2:** The age and socioeconomic results from the participants in this study.

Sample Size	*n* = 826
**Gender**	
Male	11.14%
Female	87.89%
Prefer not to answer	0.97%
**Age**	
Millennials (18–34)	19.49%
Generation X (35–54)	42.01%
Baby Boomers (55 and Over)	38.50%
**Educational Level**	
Some High School	0.00%
High School Graduate or Equivalent	2.78%
Some College	18.04%
Trade, Technical or Vocational	1.57%
Associate Degree	4.96%
Bachelor’s Degree	37.65%
Master’s Degree	24.94%
Professional Degree	4.24%
Doctorate Degree	5.81%
**Residence**	
San Luis Obispo County, CA	12.59%
California	54.24%
Other state in United States	33.17%
**Ethnicity**	
White or Caucasian	81.23%
Hispanic or Latino	6.30%
Black or African-American	0.36%
Native American or American Indian	0.00%
Asian or Pacific Islander	10.29%
Other	1.82%

**Table 3:** Results from the ANOVA for all responses. For each main factor and two-way interaction *p*-values are presented (90% confidence level). The R^2^ coefficient is indicated as well. The notation “ns” indicate a non-significant effect (*p* ≤ 0.1). Significant factors are designated as bold.

Response	R^2^	Product	Information	Insect	Product-by-Information	Product-by-Insect	Information-by-Insect
Active	0.98	**<0.0001**	ns	**0.0004**	ns	**0.0002**	ns
Adventurous	0.82	ns	**0.0951**	**<0.0001**	ns	ns	ns
Aggressive	0.70	0.8784	0.5487	ns	**0.0627**	ns	ns
Bored	0.90	**0.0202**	ns	**0.0012**	ns	**0.0689**	ns
Calm	1.00	**0.0912**	0.9460	**0.0016**	**0.0501**	**0.0636**	ns
Daring	1.00	**0.0053**	0.1004	**0.0002**	**0.0116**	**0.0391**	ns
Disgusted	1.00	0.1044	0.6751	**<0.0001**	ns	**0.0334**	**0.0006**
Enthusiastic	0.37	ns	ns	**0.0091**	ns	ns	ns
Free	0.93	0.1470	0.2935	**0.0015**	**0.0695**	ns	ns
Good	0.88	ns	ns	**<0.0001**	ns	ns	ns
Good-natured	0.64	ns	ns	**0.0001**	ns	ns	ns
Guilty	0.99	**<0.0001**	ns	**<0.0001**	ns	**0.0001**	ns
Happy	0.98	0.1416	ns	**<0.0001**	ns	**0.0334**	ns
Interested	0.99	**0.0797**	**0.0848**	**0.0075**	**0.0531**	**0.0546**	ns
Joyful	0.94	**0.0170**	ns	**0.0002**	ns	**0.0196**	ns
Loving	0.48	ns	ns	**0.0019**	ns	ns	ns
Mild	0.00	ns	ns	ns	ns	ns	ns
Nostalgic	0.94	**0.0172**	ns	**0.0001**	ns	**0.0718**	ns
Pleasant	0.85	ns	**0.0351**	**<0.0001**	ns	ns	ns
Satisfied	0.93	ns	ns	**<0.0001**	ns	ns	ns
Secure	0.98	**0.0052**	ns	**<0.0001**	ns	**0.0020**	ns
Tame	0.25	ns	ns	**0.0418**	ns	ns	ns
Understanding	0.61	ns	0.0104	**0.0008**	ns	ns	ns
Warm	1.00	**0.0291**	0.1105	**0.0032**	**0.0560**	**0.0307**	ns
Wild	0.00	ns	ns	ns	ns	ns	ns
Worried	1.00	**0.0727**	0.1388	**0.0006**	**0.0181**	**0.0577**	ns
Disgust	0.85	ns	**0.0020**	**<0.0001**	ns	ns	**0.0010**
Liking	0.99	**0.0105**	0.2742	**<0.0001**	**0.0236**	ns	ns
Willing to Try	0.99	**0.0718**	0.7186	**<0.0001**	**0.0118**	ns	ns

## Data Availability

The data presented in this study are available on request from the corresponding author. The data are not publicly available due to privacy and ethical restrictions.
